# Peripheral Ulcerative Keratitis with Pyoderma Gangrenosum

**DOI:** 10.1155/2015/949840

**Published:** 2015-10-07

**Authors:** Adrián Imbernón-Moya, Elena Vargas-Laguna, Antonio Aguilar, Miguel Ángel Gallego, Claudia Vergara, María Fernanda Nistal

**Affiliations:** ^1^Department of Dermatology, Hospital Universitario Severo Ochoa, Avenida de Orellana, Leganés, 28911 Madrid, Spain; ^2^Department of Ophthalmology, Hospital Universitario Severo Ochoa, Avenida de Orellana, Leganés, 28911 Madrid, Spain

## Abstract

Pyoderma gangrenosum is an unusual necrotizing noninfective and ulcerative skin disease whose cause is unknown. Ophthalmic involvement in pyoderma gangrenosum is an unusual event. Only a few cases have been reported, from which we can highlight scleral, corneal, and orbital cases. Peripheral ulcerative keratitis is a process which destroys the peripheral cornea. Its cause is still unknown although it is often associated with autoimmune conditions. Pyoderma gangrenosum should be included in the differential diagnosis of peripheral ulcerative keratitis. Early recognition of these manifestations can vary the prognosis by applying the appropriate treatment. We introduce a 70-year-old woman who suffered pyoderma gangrenosum associated with peripheral ulcerative keratitis in her left eye. The patient's skin lesions and peripheral keratitis responded successfully to systemic steroids and cyclosporine A.

## 1. Introduction

Pyoderma gangrenosum (PG) is an unusual necrotizing noninfective and ulcerative skin disease of unknown cause that has been included among the so-called neutrophilic dermatoses. The condition is clinically characterized by necrotic and deep ulcers that are previously preceded by inflammatory pustules [1, 2].

Under the term peripheral ulcerative keratitis (PUK), a group of inflammatory corneal diseases clinically characterized by peripherical corneal thinning, cellular infiltration, ulceration, and variable degree vasoocclusion and injection of the adjacent vascular network are included [3, 4].

Ophthalmic involvement in pyoderma gangrenosum is not a usual event. Only a few cases have been reported, from which we can highlight scleral, corneal, and orbital cases [5–10]. We report a case of PG associated with PUK in a 70-year-old woman. The patient's skin lesions and peripheral keratitis responded successfully to systemic steroids and cyclosporine A.

## 2. Case Presentation

A 70-year-old woman with a personal history of non-insulin-dependent diabetes mellitus was seen on consultation because of rapid development of an eruption consisting in several ulcerative and painful lesions located on her left leg. Initial lesions were boggy violaceous plaques with pustules that rapidly enlarged for two weeks prior to presentation. The patient was treated with oral and topical antibiotics without results. At the same time, the patient had fever and discomfort and complained of redness and pain and visual acuity decreased in her left eye.

**Figure 1:**
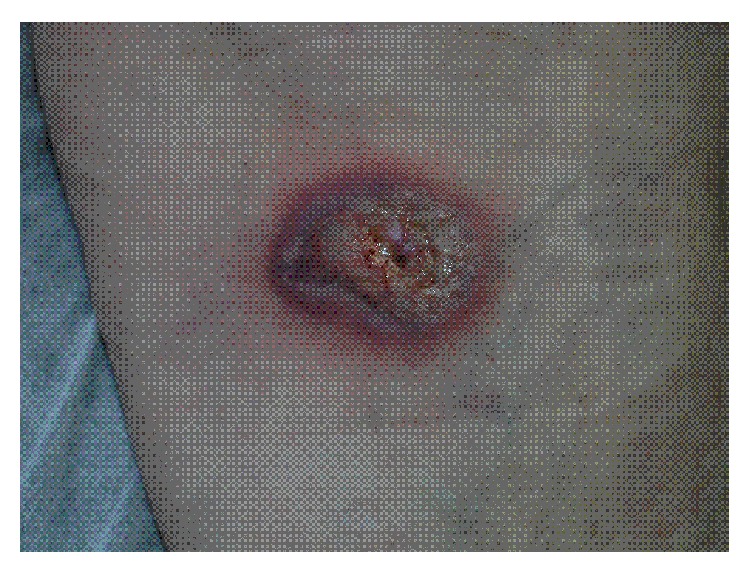
Ulcer with a necrotic base, raised border, and halo erythema on the left leg.

Cutaneous examination revealed scattered shallow ulcers with a necrotic base which were confined to the left leg. The ulcer border was raised, serpiginous, and irregular and it was surrounded by an inflammatory area of erythema (Figure 1). There were no other cutaneous findings.

**Figure 2:**
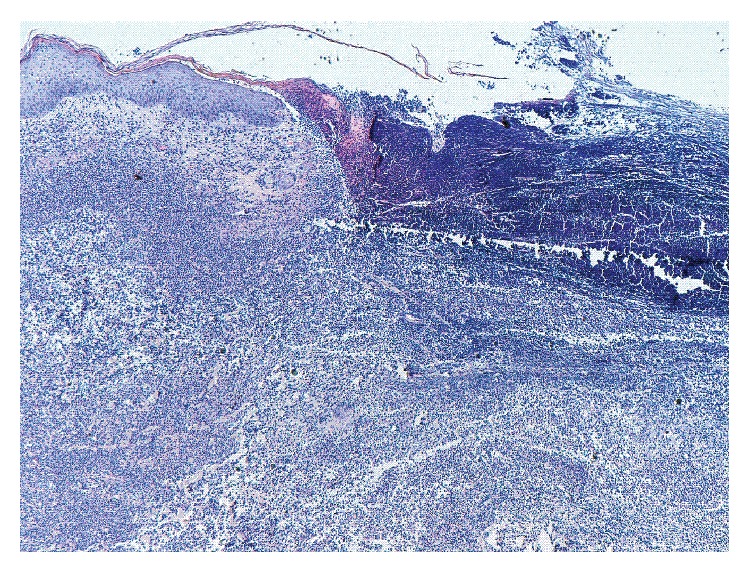
Neutrophilic abscess formation under areas of ulceration, as well as a dense inflammatory dermal infiltrate composed primarily of polymorphonuclear leukocytes (H-E ×10).

A wedge-shaped cutaneous biopsy showed neutrophilic abscess formation under areas of ulceration, as well as a dense inflammatory dermal infiltrate composed primarily of polymorphonuclear leukocytes but including occasional mature lymphocytes. No vascular involvement was observed (Figure 2). Cultures from the skin lesions were negative for fungi, mycobacteria, and bacteria.

**Figure 3:**
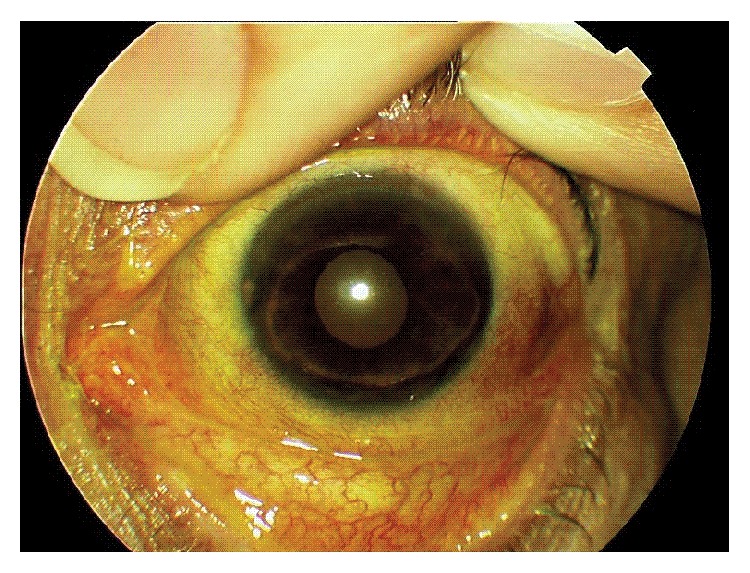
Ulceration and peripheral stromal infiltrates in the upper and lower limb (slit lamp).

Ocular examination with slit lamp revealed ulceration and peripheral stromal infiltrates in the upper and lower limb on her left eye (Figure 3).

All the following laboratory evaluations were in the normal range: biochemical parameters, complete blood cell count, white blood cell count, differential count, erythrocyte sedimentation rates, serum protein electrophoresis, quantitative serum immunoglobulins, C3 and C4 levels, antinuclear antibodies, anti-double stranded DNA antibodies, rheumatoid factor, and Venereal Disease Research Laboratory (VDRL) test. Chest X-ray examination, ultrasound examination, and thoracic-abdominal-pelvic computed tomography were all carried out although no systematic involvement was found.

The patient was diagnosed with pyoderma gangrenosum and unilateral peripheral ulcerative keratitis. Therapy was started with a course of systemic corticosteroid (prednisone 1 mg/Kg daily) obtaining a favourable response with improvement of the skin ulcers and the ocular damage after four weeks of treatment. When the dose of prednisone was reduced to 0,5 mg/Kg daily, mild relapse of the cutaneous and ocular lesions occurred, so the patient was treated with cyclosporine A 3 mg/Kg daily and prednisone 20 mg daily. After three months, the disease was totally resolved with no ocular residual damage and no new active skin lesions were detected.

## 3. Discussion

PG may appear in healthy patients or in those associated with a variety of systemic diseases. These diseases are present in more than 50% of patients. The most common is the comorbidity inflammatory bowel disease followed by rheumatoid arthritis. Others include immunologic abnormalities, hematologic disorders like monoclonal gammopathy and polycythemia vera, and hematologic malignancies like myeloma, leukemia, lymphoma, and myelodysplasia [1, 2, 5, 7–10]. This relationship supports the hypothesis that the disease may be caused due to underlying defects in the immune system such as abnormalities of cellular or humoral immunity, reduced production of macrophage inhibitory factor, disorder of chemotaxis, and phagocytosis by neutrophils and monocytes. However, a specific immune defect has not been demonstrated [1, 2, 5, 8, 10].

The skin lesions have classic appearance and evolution, starting as a papule or pustules that rapidly progress to a well defined and very painful ulcer with necrotic or mucopurulent debris at the base. The ulcer is surrounded by violaceous undermined borders and an inflammatory halo of erythema. Cutaneous lesions may be present at any site of the skin surface but mucosal membranes are usually spread. It has a propensity to appear on the lower limbs or the trunk and sometimes occurs at areas of the skin previously damaged by trauma or surgical wounds (pathergic phenomenon). The diagnosis of PG is essentially clinical and PG is considered a diagnosis of exclusion. The histopathologic findings are not specific and there are no diagnostic laboratory test markers of the disease [1, 2, 10].

PUK is a destructive process of the peripheral cornea that is often associated with autoimmune conditions including rheumatoid arthritis, Sweet syndrome, systemic lupus erythematosus, Wegener's granulomatosis, and polyarteritis nodosa. Patients may present decreased visual acuity, blindness, eye pain, redness, or irritation. The diagnosis is confirmed by slit lamp examination [3, 4, 7–10].

As with PG the aetiology of PUK is poorly understood, the postulated reasons include autoimmune reactions to corneal antigens, circulating immunocomplex deposition, vasculitis, and hypersensitivity reactions to exogenous antigens. PUK may result from humoral or cell-mediated immune mechanisms or both, causing obliterative microangiitis at the level of the limbal vascular arcades. Subsequent leakage of inflammatory cells with destructive collagenases and proteases leads to scleral inflammation and destruction [3–5, 8, 10].

There have been only seven reported cases of PUK associated with PG [5–10] (Table 1). There have been four reported cases in males and three cases in females. The age of clinical appearance varies between 30 and 78 years. PUK is usually unilateral and the left eye is the most frequently affected. There is only one case of bilateral ocular involvement [5]. Other autoimmune disorders were associated like monoarticular arthritis [6], rheumatoid arthritis [7], leukocytoclastic vasculitis [6, 7], and Graves' disease [9], as well as diseases producing immunosuppression like chronic obstructive pulmonary disease [8], diabetes mellitus [8], multiple myeloma [10], and chronic myelogenous leukemia [5]. All the patients were prescribed systemic corticosteroids. All but 1 were treated with immunosuppressive agents. These include cyclophosphamide, cyclosporine A, azathioprine, dapsone, and human intravenous immunoglobulins. All the patients had an initial adequate response but the course of the disease varied between cases with some relapsing cases. PG and PUK do not follow a parallel course.

Our patient had no other autoimmune disease associated and she had a complete response of PG and PUK with cyclosporine A and systemic corticosteroids. PG and other autoimmune disorders or diseases producing immunosuppression should be considered in the differential diagnosis of PUK. Early recognition of these manifestations can lead to the application of appropriate treatment improving the prognosis [3–10].

## Figures and Tables

**Table 1:** Description of cases reported of pyoderma gangrenosum with peripheral ulcerative keratitis (PUK). Literature review.

Authors	Gender	Age	PUK	Association	Therapy	Response to therapy
Bouchard et al. [5]	Male	37	Bilateral	Chronic myelogenous leukaemia	Systemic corticosteroids	Complete response

Bishop and Tullo [6]	Female	59	Left eye	Monoarticular arthritis	Cyclophosphamide and systemic corticosteroids	Improved but with intermittent flares of ocular diseases

Bishop and Tullo [6]	Male	56	Right eye	Leukocytoclastic vasculitis	Systemic corticosteroids	Improved but with recurrence of ocular disease

Wilson et al. [7]	Female	60	Left eye	Rheumatoid arthritis and leukocytoclastic vasculitis	Cyclosporine A and systemic corticosteroids	Improved but with intermittent flares of ocular and skin diseases

Brown et al. [8]	Male	54	Right eye	Chronic obstructive pulmonary disease and diabetes mellitus	Systemic corticosteroids and azathioprine	Complete response

Teasley et al. [9]	Female	30	Left eye	Graves' disease	Dapsone	Complete response

Fournié et al. [10]	Male	78	Left eye	Multiple myeloma	Cyclophosphamide, systemic corticosteroids, and human intravenous immunoglobulins	Complete response

## Acronyms

PG: Pyoderma gangrenosum
PUK: Peripheral ulcerative keratitis.
